# Multi-country study on the impact of a revised consensus definition of late HIV diagnosis, Europe, 2022 to 2023

**DOI:** 10.2807/1560-7917.ES.2026.31.32.2500921

**Published:** 2026-08-13

**Authors:** Peter D Kirwan, Annemarie R Stengaard, Johanna Brännström, Dominique Van Beckhoven, Ard van Sighem, Eline LM Op de Coul, Barbara Gunsenheimer-Bartmeyer, Uwe Koppe, Helena Cortes-Martins, Vítor Cabral Veríssimo, Marek Malý, Maria Wessman, Chrysa Tsiara, Georgios Ferentinos, Barbara Suligoi, Vincenza Regine, Lucia Pugliese, Sophie Grabar, Ann K Sullivan, Juliana Reyes-Urueña, Anastasia Pharris, Giorgi Kuchukhidze, Ole Kirk, Sara Croxford, Dorthe Raben, on behalf of the EuroTEST HIV Late Diagnosis Definition Working Group and EuroTEST Steering Committee

**Affiliations:** 1 MRC Biostatistics Unit, University of Cambridge, Cambridge, United Kingdom; 2 Rigshospitalet, University of Copenhagen, CHIP - Centre of Excellence in Health, Immunity and Infections, Copenhagen, Denmark; 3 Department of Infectious Diseases, Södersjukhuset, Stockholm, Sweden; 4 Department of Clinical Science and Education, Karolinska Institute, Södersjukhuset, Stockholm, Sweden; 5 Unit of Infectious Diseases and Dermatology, Department of Medicine Huddinge, Karolinska Institute, Stockholm, Sweden; 6 Department of Epidemiology and Public Health, Sciensano, Brussels, Belgium; 7 Stichting HIV Monitoring, Amsterdam, Netherlands; 8 Amsterdam institute for Immunology & Infectious diseases, Amsterdam, The Netherlands; 9 National Institute for Public Health and the Environment (Centre for Infectious Disease Control), Bilthoven, Netherlands; 10 Department of Infectious Disease Epidemiology, Robert Koch Institute, Berlin, Germany; 11 National Institute of Health, Lisbon, Portugal; 12 Directorate-General of Health, Lisbon, Portugal; 13 National Institute of Public Health, Prague, Czech Republic; 14 Statens Serum Institut, Copenhagen, Denmark; 15 National Public Health Organization, Athens, Greece; 16 Istituto Superiore di Sanità, Rome, Italy; 17 Sorbonne Université, INSERM, Institut Pierre Louis d’Epidémiologie et de Santé Publique, Paris, France; 18 Hôpital St Antoine, Département de Santé Publique, Paris, France; 19 Chelsea & Westminster NHS Foundation Trust, London, United Kingdom; 20 European Centre for Disease Prevention and Control (ECDC), Stockholm, Sweden; 21 World Health Organization Regional Office for Europe, Copenhagen, Denmark; 22 Mersey and West Lancashire Teaching Hospitals NHS Trust, Prescot, United Kingdom; 23 The members of these groups are listed under Acknowledgements

**Keywords:** Human Immunodeficiency Virus, Late diagnosis, Acquire Immune Deficiency Syndrome, Seroconversion

## Abstract

**BACKGROUND:**

With improvements in test sensitivity and testing frequency, more people in Europe are diagnosed during the HIV acute/seroconversion phase, when their CD4^+^ T-cell count can be temporarily low.

**AIM:**

We piloted a revised definition of late HIV diagnosis for surveillance which considers laboratory markers, evidence of HIV test negativity in the past year and clinical evidence of recent HIV acquisition, to better distinguish between people diagnosed late and those diagnosed during the acute/seroconversion phase.

**METHODS:**

We collected pseudonymised HIV diagnosis records for 2022 and 2023 from nine European countries (Belgium, Czechia, Denmark, Germany, Greece, Italy, the Netherlands, Portugal and Sweden). We applied the revised definition to reclassify those with evidence of recently acquired HIV as 'not late' and calculated the corresponding correction factor by country and demographic group. Individuals with a previous diagnosis were excluded from the analysis.

**RESULTS:**

Among 10,220 individuals with a CD4^+^ T-cell count at diagnosis, 56% (5,689/10,220) had a CD4^+^ T-cell count < 350 cells/µL or an AIDS-defining event at diagnosis. Applying the revised definition reduced the late diagnosis proportion from 56% to 50%, a correction factor of 9% (535/5,689). The correction factor ranged between 3% and 25% across countries and was higher for younger ages and for gay, bisexual and other men who have sex with men, compared with other groups.

**CONCLUSIONS:**

Without reclassification, late HIV diagnosis proportions are overestimated. We provide recommendations and validity considerations for countries intending to use the revised definition.

**Key public health message**

**What did you want to address in this study and why?**

People diagnosed very soon after acquiring HIV can have a temporarily low CD4^+^ T-cell count, making it appear as if they had been living with HIV for a long time before diagnosis. As a result, the standard, CD4^+^ T-cell -based, European measure of 'late HIV diagnosis' may misclassify some people. We investigated how much difference a revised definition of late HIV diagnosis makes across nine European countries.

**What have we learnt from this study?**

Using the revised definition reduced the proportion classified as late diagnoses from 56% to 50%. The size of this correction varied between countries, from 3% to 25%. The correction was largest in younger people and gay, bisexual and other men who have sex with men, where up to 28% of those initially classified as late were probably diagnosed during acute infection.

**What are the implications of your findings for public health?**

The revised definition provides countries with a more accurate picture of where delayed HIV testing remains a problem. Even after correction, half of the people in this study were still diagnosed late. To use this measure routinely, countries need improved routine data collection on markers indicating a recently acquired HIV infection, and agreed clinical criteria for when a diagnosis should count as recent or late.

## Introduction

A significant delay between HIV acquisition and diagnosis has implications both for the individual and for public health. For the individual, later engagement with healthcare services entails delayed access to anti-retroviral therapy (ART), a higher prevalence of comorbidities, and increased risk of mortality [1-3]. Meanwhile, for public health, a delayed diagnosis implies missed opportunities for HIV testing and ART initiation to eliminate the risk of onward transmission [4].

In Europe, biomarkers for HIV diagnosis and disease progression, including HIV antibody/antigen (p24 antigen) combination tests, CD4^+^ T-cell counts, and HIV viral load, are routinely used at the point of diagnosis for clinical assessment of immune function and disease staging [5]. Following HIV acquisition, each of these biomarkers changes with time, with CD4^+^ T-cell counts declining steadily after seroconversion and routinely used to infer time since HIV acquisition [6]. An adult diagnosed with a CD4^+^ T-cell count of 350 cells/μL, and outside the acute infection phase, is likely to have been living with HIV for 3–5 years on average, although this varies across individuals according to factors such as age, ethnicity and likely mode of HIV transmission [7-9]. While CD4^+^ T-cell thresholds are no longer applied to inform treatment initiation [10], baseline CD4^+^ T-cell testing at diagnosis remains of clinical importance, and an important quality indicator in the European standards of HIV care and prevention [5].

In 2010, 'late HIV diagnosis' was defined by a European working group under the EuroTEST Initiative as a CD4^+^ T-cell count < 350 cells/μL blood or an AIDS-defining event at diagnosis [11]. This consensus definition was widely adopted in Europe and is a standard metric used by the European Centre for Disease Prevention and Control (ECDC) and the World Health Organization (WHO) Regional Office for Europe for surveillance purposes [5,12]. Late HIV diagnosis is also used to evaluate HIV testing programmes, since a reduction in the late diagnosis rate (after controlling for factors such as migration and transmission dynamics) implies a trend towards earlier testing [13,14]. With Europe aiming for the elimination of HIV transmission by 2030 [12], monitoring of late HIV diagnosis is increasingly relevant for pinpointing the groups most at risk of missing this target [2].

In recent years, increases in testing frequency among gay, bisexual and other men who have sex with men (gbMSM), and the widespread use of more sensitive fourth-generation HIV tests have resulted in more people in Europe being diagnosed during the acute (or seroconversion) phase of infection, when their CD4^+^ T-cell count can be temporarily low [5,15]. These individuals will, according to the 2010 consensus definition, be misclassified as having been diagnosed late with HIV. This issue was observed first in country-level data [16-18] and led to a revised definition of late HIV diagnosis being published in 2022 [15]. In addition to CD4^+^ T-cell counts and AIDS-defining events, the revised definition considers other biomarkers of recent HIV acquisition to better distinguish between people diagnosed late and those diagnosed during the acute phase of infection (Box). The resulting adjustment for late HIV diagnosis is termed a 'correction factor'.

Box **Revised definition of late HIV diagnosis published in 2022 [15]**

Late HIV diagnosis is defined as a person first diagnosed with HIV either with a CD4^+^ T-cell count < 350 cells/μL or with an AIDS-defining event regardless of the CD4^+^ T-cell count. People with evidence of recent acquisition (i.e. being diagnosed during seroconversion) should be reclassified as 'not late'. Evidence of recent acquisition should be considered hierarchically; the person must have:

- laboratory evidence of recent acquisition (recent infection testing algorithm, p24 antigen, or a positive PCR with a negative/indeterminate antibody test)^a^;
- or a last negative HIV test within 12 months before HIV diagnosis;
- or diagnosis during the acute stage based on clinical evidence (e.g. seroconversion illness).

People with evidence of having been previously diagnosed, either abroad or elsewhere, should be excluded from the calculation of the proportion of the population diagnosed late.

^a^ A positive PCR with negative/indeterminate antibody test was not part of the published definition but added here for the purpose of operationalising the definition as part of this implementation study. We also recommend avoiding p24 positivity alone to reclassify people with AIDS defining illnesses, or corroborating p24 antigen positivity with a negative/indeterminate antibody test, because p24 antigen levels may increase in the AIDS phase.

So far the revised definition has been adopted by relatively few countries in Europe [18-20], so there is limited experience to support countries with differing availability of the recent HIV acquisition markers.

We aimed to pilot the revised late HIV diagnosis definition in European countries with varied epidemic profiles and data collection systems. Our specific objectives were: (i) to generate country-specific estimates of correction factors and apply these to surveillance data; (ii) to produce updated estimates of late HIV diagnosis in the participating countries; and (iii) to propose analysis approaches and validity considerations for countries with differing availability on the various markers of recent HIV acquisition.

## Methods

### Study design

This cross-sectional study was designed and run by the EuroTEST study coordinating centre (EuroTEST secretariat in collaboration with the EuroTEST HIV Late Diagnosis Definition Working Group). The study protocol is included in the Supplement.

### Setting

We invited to participate those countries from the WHO European Region that: (i) expressed interest in participating in the pilot via a 2022 country survey on availability of data to adjust for recent infection and (ii) had country level data available on at least one of the three markers of recent infection or (iii) reported > 30 cases of 'acute infection' to ECDC as part of the annual European HIV surveillance data call for 2022. The nine countries included were (in alphabetical order): Belgium, Czechia, Denmark, Germany, Greece, Italy, the Netherlands, Portugal and Sweden.

### Participants

We included all individuals 15 years or older and newly diagnosed with HIV in one of the participating countries during calendar years 2022 and 2023. People with evidence of having been diagnosed previously ('previously known HIV positives') either abroad or already recorded in national systems were removed from national datasets before submission to EuroTEST. Data were pseudonymous, with no personal identifiers collected. We chose 2022 as the starting year to minimise the impact of COVID-19 disruptions on HIV testing.

### Data sources

The country study coordinator extracted, on behalf of the participating country, national datasets from existing national cohorts and surveillance registries and submitted them to the EuroTEST study coordinating centre via secure file transfer protocol (FTP) between August and October 2024. A pre-defined Excel reporting template with variable definitions was provided and data were validated using a set of validation criteria (e.g. variable coding, date format). Data queries were followed up with the country study coordinator.

### Variables

The collected variables and their definitions were aligned with the existing ECDC/WHO European level surveillance reporting format (The European Surveillance System - TESSy [21]), to reduce workload as much as possible – except for the new variables on recent HIV acquisition that were being piloted. This would also allow operationalising this definition more broadly following this pilot. The full list of collected variables and coded values is included in the study protocol in the Supplement. Briefly, these were: age at time of HIV diagnosis (exact age or age category), gender (female, male, other, transgender, unknown), route of HIV transmission (sex between men (gbMSM), sex between men and women, people who inject drugs (PWID), other, unknown), date of HIV diagnosis, date of AIDS diagnosis, date of death, first CD4^+^ T-cell count, date of first CD4^+^ T-cell count, country of birth/region of origin, and setting of first positive HIV test. Heterosexual transmission was subdivided into female/male on the basis of gender. Month and year were accepted for the date variables if exact dates were not available. We defined CD4^+^ T-cell count at diagnosis and AIDS at diagnosis as a CD4^+^ T-cell count or AIDS diagnosis dated within 90 days of HIV diagnosis, respectively. The 90-day threshold is common in surveillance analyses to capture clinical status close to diagnosis but allow for routine delays in laboratory testing and reporting.

Three variables representing markers of recent HIV acquisition were also collected for each diagnosis: laboratory evidence, a recent negative HIV test, and clinical evidence. Multiple responses could be reported for each marker. Laboratory evidence was based on a recent result from the recent infection testing algorithm (RITA), a positive p24 antigen, or a positive PCR with a negative/indeterminate antibody test. Negative test evidence was a last negative HIV test within 12 months before HIV diagnosis. Note that whereas the revised definition uses 'within 12 months', some countries only had a 6-month history available, providing a more conservative estimate. Clinical evidence was seroconversion illness, or evidence of primary HIV infection (defined as high-risk exposure within the previous 6 weeks and detectable virus in plasma (p24 antigen and/or HIV RNA) and/or evolving anti-HIV antibody reactivity (negative or indeterminate to positive), with or without clinical symptoms [22]). All markers could be reported as 'non-recent' or 'missing' to distinguish between a negative result and missing data. No standardisation procedures were applied to the results obtained from avidity assays or clinical evidence of recent acquisition to account for differences between the included countries.

### Analysis

For each country, we applied the revised definition of late HIV diagnosis and reclassified people with evidence of recent HIV acquisition as 'not late'. A binary reclassification variable (Yes/No) was derived to record the evidence of recent acquisition for each diagnosis. In case of conflicting evidence, we applied rules which were agreed via working group discussion: Laboratory evidence and a negative test within 12 months were both reasons for reclassification, even in the presence of conflicting information; Clinical evidence was considered a more subjective marker and so was only valid as evidence for reclassification in the absence of a non-recent avidity result.

The same analysis was then conducted with pooled data from all countries, allowing trends in smaller demographic groups and diagnosis settings to be investigated. Late diagnosis and correction factor estimates were discussed with each country and the working group to gather experiences with country-specific data collection practices and understand potential sources of bias.

### Statistical methods

Laboratory evidence, last negative test and clinical evidence were considered hierarchically in accordance with the published definition [15]. Estimated late diagnosis correction factors were calculated as: (number reclassified)/(number with CD4^+^ T-cell count < 350 cells/μL) and evaluated by country and demographic factor.

We demonstrate the use of multiple imputation to construct correction factor ranges. For this we used chained equations to impute missing values of age group, route of transmission, country of origin, a binary late diagnosis status, and the binary reclassification variable, based on the published definition. For consistency between countries, imputation was performed on the basis of the other observed analysis variables. Missing values of the binary reclassification variable were imputed for individuals with an imputed late diagnosis only. We used 150 replicates for each country (chosen to be sufficient based on the fraction of missing information [23]) and estimated for each replicate the correction factor based on the non-missing and the imputed values for late diagnosis. The specification used for each variable is included in Supplementary Table S1.

The range of correction factors over all 150 replicates was compared with the correction factors estimated using the data as reported (i.e. with missing values). For countries that used avidity tests (Czechia, Germany and Italy), we additionally compared with the correction factors among only those diagnoses with an avidity result (recent/not recent) available.

All analyses of individual-level data were performed using Stata v18.5 (College Station, United States). Figures were generated from aggregated data using R v.4.5.1 (R Core Team, Vienna, Austria).

## Results

### Descriptive analyses

The HIV surveillance reporting mechanisms varied by country, and reporting was done by clinicians or laboratories or both. The HIV surveillance systems were based on clinical and/or laboratory reporting (Belgium, Czechia, Denmark, Italy, Germany, Greece, Portugal), or as part of a clinical network or cohort (the Netherlands, Sweden) (Table 1). Clinical evidence of recent HIV acquisition was determined in a variety of ways depending on national HIV testing guidelines, reporting systems and clinical practice; no two countries collected the same information (Table 1).

**Table 1:** Reporting mechanisms for HIV surveillance data, sources of evidence and availability of guidance for reporting clinical evidence of recent HIV acquisition, nine study countries, 2022 and 2023

	Reporting system	Laboratory evidence	Last negative test evidence	Clinical evidence	Clinical evidence guidance to clinicians
Belgium	Clinics, laboratories	p24	Clinical testing history (6 months)	Clinical stage, clinical judgement, symptoms	No
Czechia	Clinics, laboratories	Avidity assay, p24	Self-report and clinical testing history (12 months)	Clinical judgement, symptoms	No
Denmark	Clinics	None	Self-report (6 months)	Clinical stage, clinical judgement, symptoms, with secondary confirmation^a^	No
Germany	Clinics, laboratories	Avidity assay (BioRad)	Self-report and clinical testing history (12 months)	Clinical judgement, may be conflated with last negative test	No
Greece	Clinics, laboratories	None	Clinical testing history (6 months)	Clinical stage, clinical judgement, seroconversion symptoms	No
Italy	Clinics	Avidity assay, p24	Clinical testing history (12 months)	Clinical stage, clinical judgment, symptoms	No
The Netherlands	Clinical network	p24	Self-report and clinical testing history (6 months)	Symptoms, recent risk, with secondary confirmation^a^	No
Portugal	Clinics, laboratories	None	Self-report and clinical testing history (12 months)	Clinical stage, with secondary confirmation^a^	No
Sweden	Nationwide cohort (outpatient clinics, laboratories)	None	Negative serology (12 months)	Hybrid marker based on clinical judgement plus laboratory indication^b^	Yes (hybrid definition)

^a^ Secondary confirmation is employed when there are conflicting signals in the data: public health teams will reconfirm clinical evidence with diagnosing centres.

^b ^Hybrid definition used in Sweden: An infection can be considered to be recent if a person presents with seroconversion illness (based on clinical judgement of symptoms and medical history) and/or evidence of primary HIV infection (high-risk exposure within previous 6 weeks and (i) detectable virus in plasma (p24 antigen and/or HIV-RNA) and/or (ii) evolving anti-HIV antibody reactivity (negative or indeterminate to positive) – with or without clinical symptoms). Aligned with the European AIDS Clinical Society [22].

Overall, across the nine countries in the study, a total of 15,924 new adult HIV diagnoses were recorded during 2022 and 2023. The availability of CD4^+^ T-cell count data and of the three markers of recent HIV acquisition (laboratory evidence, last negative test evidence and clinical evidence) differed by country, year of diagnosis and demographic group. The number of new HIV diagnoses and availability of these markers by country, year of diagnosis and demographic group are appended in Supplementary Table S2. Availability of CD4^+^ T-cell count within 90 days of HIV diagnosis ranged from 30% in Germany to 96% in Denmark and Sweden. Most countries consider that diagnoses with CD4^+^ T-cell counts reported are broadly representative of all new diagnoses, with minimal bias in CD4^+^ T-cell data collection mechanisms.

Reporting of recent HIV acquisition markers was more varied: only five of nine countries collected laboratory evidence (Table 1). Including non-recent responses, variable completeness by country ranged from 15% to 100% for laboratory evidence, 3% to 22% for last negative test, and 3% to 100% for the clinical evidence marker. One country (Czechia) undertook centralised avidity testing of all new HIV diagnoses. Elsewhere, countries reported that laboratory testing (avidity or p24) was more likely to be undertaken by larger clinics in urban areas. Countries reported better completeness of last negative test and clinical evidence variables in larger clinics with established reporting mechanisms. The availability of a last negative test within 6–12 months reflects testing patterns and was notably higher among younger people, transgender people and gbMSM. Reporting of last negative test information was often based on self-reported data; only Sweden used negative serology information (Table 1). Route of HIV transmission was correlated with age, gender, site of test and region of origin. Supplementary Figure S1 provides bar charts for each demographic/test characteristic.

### Re-classification hierarchy

**Figure 1:**
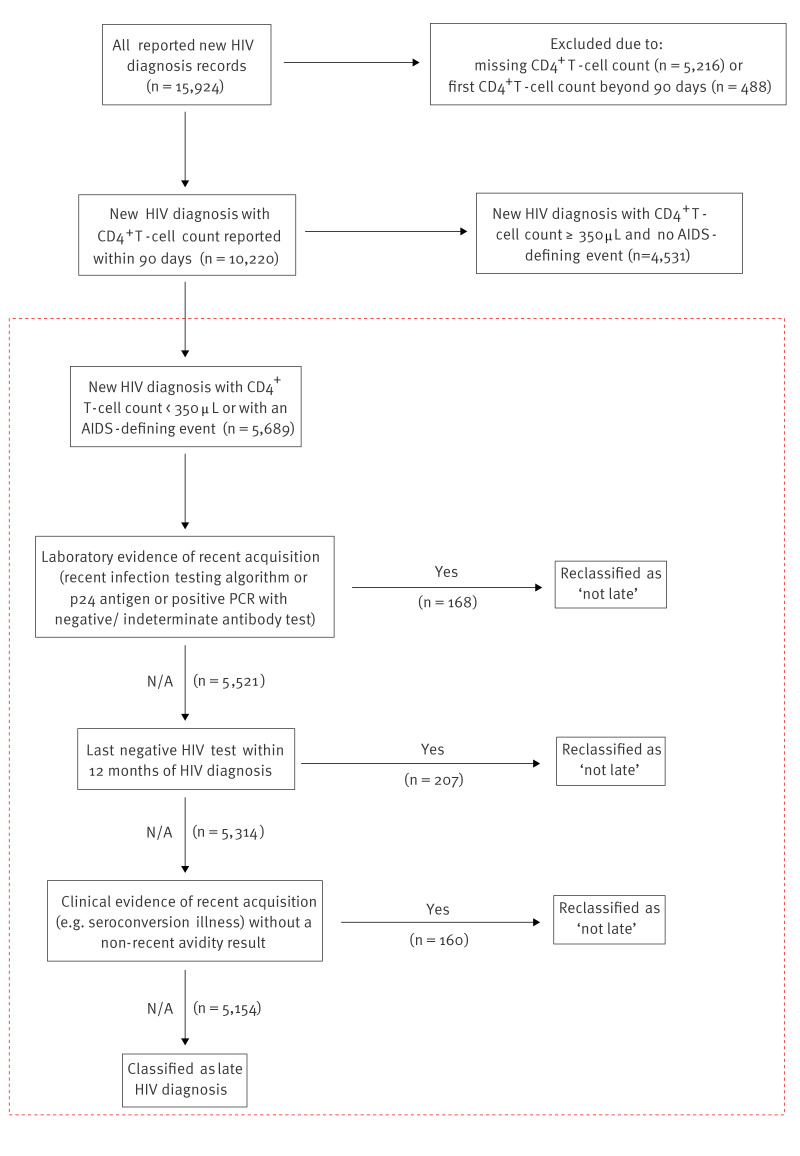
Late HIV diagnosis reclassification flowchart corresponding to evidence hierarchy, nine study countries, 2022 and 2023 (n = 15,924 new diagnosis records)

Of the 15,924 new diagnoses, 5,216 were excluded from the late diagnosis analysis because CD4^+^ T-cell counts were missing, and 488 were excluded because CD4^+^ T-cell counts were dated more than 90 days after diagnosis. Among the remaining 10,220 diagnoses, 5,689 had a CD4^+^ T-cell count < 350 cells/μL or an AIDS-defining event within 90 days (Figure 1). Three markers of recent HIV acquisition were collected for each diagnosis, with possibility for conflicting evidence. The possible combinations of evidence are provided in Supplementary Table S3, ordered according to the hierarchy of the published definition and with details for items with conflicting evidence which were agreed via working group discussion. Among the 342 late diagnoses with clinical evidence of recent HIV acquisition reported, 38% (130/342) had an avidity test reported and 8% (27/342) were over-ruled by a non-recent avidity result. For the number of late diagnoses reclassified in each evidence category, we refer to Supplementary Table S3. A flowchart corresponding to this hierarchy is shown in Figure 1, and the specific markers reported by each country are appended in Supplementary Table S4.

Nine study countries: Belgium, Czechia, Denmark, Germany, Greece, Italy, the Netherlands, Portugal and Sweden. The dotted red frame indicates the reclassification procedure.

### Re-classification by country

Overall, 56% (5,689/10,220) of people diagnosed during 2022 and 2023 had a CD4^+^ T-cell count < 350 cells/μL or an AIDS-defining event within 90 days of diagnosis and were classified as late according to the previous consensus definition. Of these, 535 had evidence of recent HIV acquisition: 168 had laboratory evidence, 237 had testing history evidence and 304 had clinical evidence (multiple forms of evidence could be reported for each individual) (Table 2).

**Table 2:** Late HIV diagnosis reclassification table demonstrating implementation of revised definition, nine study countries, 2022 and 2023 (n = 10,220 diagnosis records with a CD4^+^ T-cell count)

	CD4^+^ T-cell count within 90 days	CD4^+^ T-cell count < 350 cells/μL	Late diagnosis proportion in % (previous definition)	Reason for reclassification				Late diagnosis proportion in % (revised definition)	Correction factor (%)
				Laboratory evidence	Last negative test evidence	Clinical evidence	At least one		
Overall	10,220	5,689	56	168	237	304	535	50	9
Country									
Belgium	859	374	44	24	28	78	94	33	25
Czechia	420	210	50	6	2	5	6	49	3
Denmark	213	137	64	0	7	6	12	59	9
Germany	1,588	826	52	29	36	80	111	45	13
Greece	562	332	59	0	7	6	13	57	4
Italy	4,141	2,466	60	60	72	55	149	56	6
The Netherlands	748	364	49	49	35	47	79	38	22
Portugal	1,377	797	58	0	36	20	53	54	7
Sweden	312	183	59	0	14	7	18	53	10
Year of HIV diagnosis									
2022	4,978	2,753	55	80	98	149	247	50	9
2023	5,242	2,936	56	88	139	155	288	51	10
Age group (years)									
15–24	842	315	37	17	23	37	60	30	19
25–34	2,884	1,302	45	50	113	105	197	38	15
35–44	2,625	1,483	56	39	47	54	111	52	7
45–54	2,058	1,297	63	29	29	49	87	59	7
55–64	1,343	939	70	28	20	50	65	65	7
≥ 65	466	352	76	5	5	9	15	72	4
Gender									
Female	2,319	1,413	61	19	25	39	74	58	5
Male	7,838	4,254	54	147	209	263	457	48	11
Transgender	54	19	35	2	3	2	4	28	21
Unknown	6	3	50	0	0	0	0	50	0
Route of HIV transmission									
gbMSM	4,497	2,068	46	94	167	175	320	39	15
Hetero (female)	2,027	1,226	60	17	22	36	67	57	5
Hetero (male)	2,105	1,389	66	35	23	52	82	62	6
PWID	402	219	54	4	6	7	13	51	6
Other	40	18	45	0	0	0	0	45	0
Unknown	1,124	762	68	18	17	34	51	63	7
Site of test^a^									
Community-based testing programme	229	84	37	1	8	2	9	33	11
Accident and emergency department	257	188	73	0	5	9	13	68	7
Infectious disease clinic	249	147	59	2	3	4	8	56	5
Primary healthcare	615	291	47	21	19	24	39	41	13
Sexual health or STI clinic	351	116	33	10	20	11	26	26	22
Other hospital setting	580	443	76	21	11	23	38	70	9
Other setting	308	152	49	0	10	3	11	46	7
Undetermined or unknown	7,631	4,268	56	113	161	228	391	51	9
Region of origin									
Same as country of report	5,616	3,086	55	99	124	177	301	50	10
Central Europe^b^	454	253	56	10	9	13	23	51	9
Eastern Europe^b^	674	410	61	7	11	11	21	58	5
Western Europe^b^	230	99	43	9	7	18	22	33	22
North Africa and Middle East	247	113	46	2	5	6	8	43	7
Sub-Saharan Africa	1,453	895	62	12	26	35	60	57	7
Latin America and Caribbean	975	477	49	17	36	22	61	43	13
South and South-East Asia	293	188	64	10	9	14	23	56	12
Abroad but subcontinent unknown	73	46	63	1	6	3	7	53	15
Unknown	205	122	60	1	4	5	9	55	7
AIDS at diagnosis									
Yes	1,685	1,685	100	23	21	22	52	97	3
No	8,535	4,004	47	145	216	282	483	41	12

gbMSM: gay, bisexual and other men who have sex with men; PWID: people who inject drugs; STI: sexually transmitted infection.

^a^ Site of testing was only available in four countries (Czechia, Denmark, the Netherlands and Portugal).

^b ^If different from the country of report.

After reclassification using the hierarchy defined above, the late diagnosis proportion was reduced from 56% to 50%, with an overall correction factor of 9% (535/5,689) (Table 2). In Supplementary Figure S2, we make available the proportion diagnosed late before and after re-classification, and the correction factor by country, which ranged between 3% and 25%.

### Pooled re-classification

**Figure 2:**
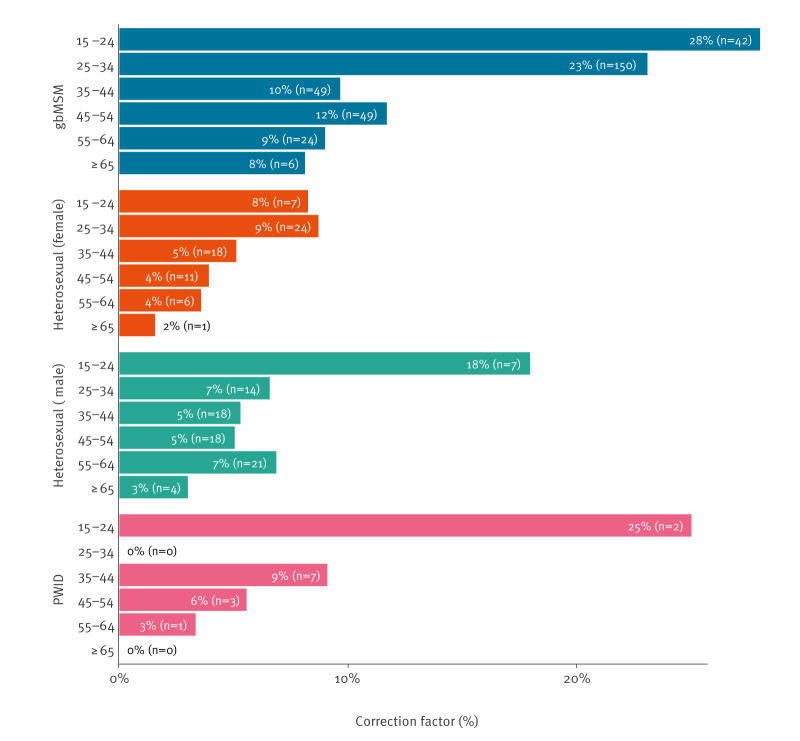
Correction factor by route of HIV transmission and age group, nine study countries combined, 2022 and 2023 (n = 10,220 diagnosis records with a CD4^+^ T-cell count)

In pooled results from all countries, the correction factor was higher for gbMSM (15%) than for other transmission routes (0–7%) (Table 2). The correction factor was also higher for younger individuals than for older adults (19% for those aged 15–24 years vs. 4% for those 65 years and older at diagnosis). This age-related pattern was consistent across all exposure groups, with gbMSM aged 15–24 years showing the highest correction factor of all groups (28%) (Figure 2).

gbMSM: gay, bisexual and other men who have sex with men; PWID: people who inject drugs.

Data labels are the correction factor (%) and number of diagnoses (n). Nine study countries: Belgium, Czechia, Denmark, Germany, Greece, Italy, Netherlands, Portugal and Sweden.

Four countries (Czechia, Denmark, Netherlands and Portugal) were able to report information on site of HIV test. While the numbers were small, as shown in Table 2, the correction factor was highest in sexual health and sexually transmitted infection (STI) clinics (22%), followed by primary healthcare (13%) and community-based testing sites (11%). In Supplementary Figure S3 we append bar charts for each country. 

The correction factor was 10% among people who originated in the reporting country, higher among migrants from western Europe (22%) and Latin America and the Caribbean (13%), and lower among migrants from eastern Europe (5%), North Africa and the Middle East (7%) and Sub-Saharan Africa (7%) (Table 2).

### Imputation of lower and upper bounds

**Figure 3:**
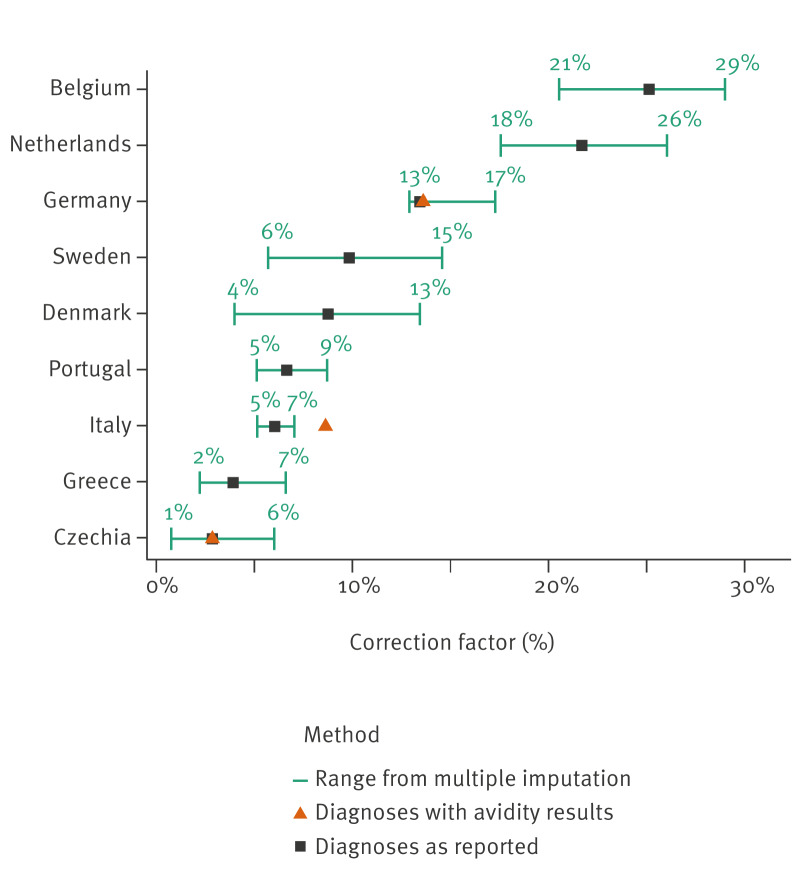
Imputed correction factor ranges from multiple imputation compared with correction factors from diagnoses with laboratory evidence available and from the observed-data analysis with diagnoses as reported, nine study countries, 2022 and 2023 (n = 15,924 new diagnosis records)

Figure 3 shows the correction factor range estimated by multiple imputation, the correction factor estimated by restricting to diagnoses with avidity results, and the correction factor estimated using the diagnoses as reported (i.e. with missing values), by country. The width of the ranges reflects the level of uncertainty and ranges were wider in countries with a higher proportion of missing data. Supplementary Table S5 provides the proportion of values imputed for each variable. In all countries, the ranges estimated by multiple imputation included the estimated correction factor for diagnoses as reported. In countries with avidity results available (Czechia, Germany and Italy), the avidity-only correction factor was the same as or higher than the correction factor for diagnoses as reported.

Data labels are the upper and lower ends of the range estimated from multiple imputation. Points are the correction factors from all diagnoses as reported or only those diagnoses with avidity results available. Avidity testing was only performed in Czechia, Germany and Italy.

## Discussion

We applied the revised late HIV diagnosis definition in nine European countries, operationalising a suitable hierarchy of conflicting evidence. We found that the late HIV diagnosis proportion without correction is overestimated, and this misclassification affects all countries, routes of transmission and age groups. The extent of misclassification was 9% on average but ranged across countries from 3% to 25%, and was as high as 28% among young gbMSM.

Our findings are consistent with country-level analyses from Belgium and the United Kingdom, which demonstrated that the previous consensus definition can overestimate late diagnosis in settings where frequent testing and ascertainment of recent acquisition are more common [16,18]. They also align with more recent national analyses and surveillance outputs from Italy and Denmark, where the updated definition is used for annual reporting [19,20].

In our study, the highest correction factors were found in Belgium, the Netherlands and Germany, with up to a quarter of late HIV diagnoses incorrectly classified based on the previous definition. Lower correction factors were seen in Czechia, Greece and Italy. Correction factors were affected by the completeness of the variables used for reclassification, but also reflect the characteristics of each country’s epidemic: repeat testing patterns, access to testing, pre-exposure prophylaxis (PrEP) policies, and perceptions of at-risk sexual behaviours in different groups, e.g. older people and heterosexual men and women, all of which vary across settings and influence the likelihood of diagnosis during the acute stage [24,25].

In pooled country data, the highest correction factors were observed for gbMSM and younger individuals from all transmission groups. These individuals may be targeted for repeat testing and are consequently more likely to be diagnosed during the acute or seroconversion stage [24]. Correction factors were much lower among heterosexual men and women, people who inject drugs, and non-Western European migrants. These groups are known to be less likely to undergo repeat HIV testing and may encounter regulatory barriers to testing or avoid formal healthcare services [24,26].

Where data were available, correction factors were highest for diagnoses made in sexual health clinics, community testing programmes and primary healthcare. After correction, these settings also had the lowest proportion diagnosed late. There is evidence that community testing programmes contribute to earlier diagnoses among gbMSM [13], and sexual health clinics may undertake partner notification which provides the opportunity for earlier diagnosis [27]. Elsewhere, primary healthcare settings have been identified as promising settings for increased access to earlier testing [24,25], and the ECDC’s integrated HIV, hepatitis B and C testing guidance encourages geographically targeted routine testing [24].

Information used for reclassification was collected opportunistically, so the estimated correction factors represent a lower bound on the true correction factor. For a complete data scenario, we explored restricting the analysis to only those diagnoses with avidity results available, since these provide a designation of recent/non-recent HIV acquisition which is more reliable during the acute stage [28]. The avidity-only correction factors were slightly higher than the correction factors for all diagnoses, however, these tests were only used in a third of the countries in our study and there may be biases in how testing is offered. A correction factor range could better reflect the potential for re-classification, and we demonstrated the use of multiple imputation to estimate ranges accounting for missing data. Imputed ranges were consistent with estimates from the observed data and narrower in countries with over 50% data completeness in the CD4^+^ T-cell count, laboratory evidence and clinical evidence indicators, which serves as a further justification for European countries to improve completeness of these key variables for surveillance.

From a public health perspective, opting to not reclassify diagnoses made during the acute stage will inflate late diagnosis indicators used for national surveillance, and HIV testing programmes may appear less effective as a result, particularly in groups with frequent repeat testing [18]. For service planning, uncorrected late diagnosis estimates may overstate the burden attributable to delayed testing and under-recognise the need for pathways for acute infection diagnosis, partner notification and rapid linkage to care. This distinction is more important for surveillance and programme planning than for individual treatment decisions, since both recently acquired and genuine late diagnoses should be offered prompt ART initiation and clinical follow-up [10].

Although we corrected for misclassification, half of the newly diagnosed individuals in our study remained classified as late diagnoses, with an elevated risk of adverse health impacts [1]. Improving the availability and acceptability of HIV testing by addressing structural barriers to testing, such as cost, access, complicated consent requirements and legal barriers, and challenging HIV-related stigma can help to reduce late diagnosis [29,30]. Late HIV diagnosis was as high as 58% among migrants from eastern Europe and Sub-Saharan Africa. A number of specific barriers to testing for migrants in Europe have been identified, including communication issues, lack of trust in healthcare systems and fear of the implications of a positive result [31].

The high proportion of missing CD4^+^ T-cell counts and reclassification variables for certain countries, particularly Germany, Greece and Belgium, might affect the reliability of our late diagnosis estimates, particularly if these data are not missing at random, or if linkage processes introduce selection bias. Germany contributed the greatest number of HIV diagnosis in our study, and 70% of these diagnoses had missing CD4^+^ T-cell information. In Germany, laboratories notify HIV diagnoses directly to the Robert Koch Institute, with some laboratories sharing residual blood samples for centralised avidity testing. Meanwhile, CD4^+^ T-cell counts are reported separately by clinicians, and these reports may be delayed or records not linked. The reasons why these data are missing are therefore largely procedural, and the degree of selection bias is expected to be minimal. We used imputation to illustrate the uncertainty introduced by missing data, with wider variability in the estimated correction factors where completeness was lower. Countries may consider adjusting for these factors at a local level by considering a wider range of variables when imputing missing data, or by applying specific inclusion criteria tailored to the national context [19].

Our study was limited to countries with reclassification markers available at a national level, so the data were not a representative sample of HIV diagnoses across Europe. Nonetheless, data were collected for diagnoses across a variety of settings and demographic groups, revealing a wide range in the estimated correction factors.

We collected all available diagnosis records, CD4^+^ T-cell counts, and both missing and complete data on all three recent acquisition markers so that biases related to missing data could be investigated. Although all of the countries undertook deduplication to remove previous diagnoses already recorded in national systems, classification of previous diagnoses abroad is typically based on clinic reporting, and individuals would not have been excluded from our analysis if this was misreported. We did not collect ART information and were therefore unable to exclude CD4^+^ T-cell counts after ART initiation; however, CD4^+^ T-cell counts were only considered within 90 days of HIV diagnosis.

There are specific limitations with the interpretations of each marker. Firstly, avidity assays and testing protocols were not standardised across countries, for example, Czechia and Germany use centralised avidity testing, whereas Italy sources test results from multiple laboratories. Additionally, while algorithms are usually applied to reduce false recency rate, reliability concerns have been noted with certain assays [32]. Secondly, the p24 antigen assay may be positive during not only the early but also the late phase of HIV infection [33]. We confirmed that none of the 42 (2%) individuals reported with AIDS-defining illness at diagnosis were reclassified solely on the basis of a p24 antigen assay. For last negative test evidence, this was often self-reported and could be viewed as unreliable if not corroborated. Finally, clinical evidence of recent HIV acquisition is not yet standardised and varied substantially across diagnosis settings and countries. Compared with Sweden’s hybrid clinical definition, the symptom/judgement-based reporting used elsewhere is more subjective and likely to be under-reported, and may therefore underestimate recent diagnoses.

We operationalised the reclassification evidence hierarchy which considers laboratory evidence, followed by last negative tests, followed by clinical evidence. Aligning with the overall hierarchy proposed in the 2022 definition is recommended, however some countries may wish to apply different hierarchies based on local reporting practices. In our implementation, we treated 'N/A' and 'missing' as operationally equivalent for the last negative test and clinical evidence markers, and excluded clinical evidence where an avidity assay indicated 'not recent'. We excluded CD4^+^ T-cell counts taken after ART initiation from our late diagnosis calculations.

Countries using avidity assays should apply a suitable recent infection testing algorithm to mitigate false recency in this biomarker, and may also wish to avoid using p24 positivity alone to reclassify people with AIDS-defining illnesses, or to corroborate p24 antigen positivity with a negative/indeterminate antibody test. More generally, a standardised clinical definition of recent HIV acquisition would improve comparability between countries.

## Conclusion

This study demonstrates that applying a correction to the proportion of late HIV diagnoses provides a more accurate picture of its magnitude. Such adjustment is particularly relevant for younger individuals and gbMSM, where a substantial fraction of cases classified as late represent acute infections with temporarily low CD4^+^ T-cell counts. Accounting for this distinction is critical to guiding strategies that prioritise early testing and prompt diagnosis, thereby helping to reduce onward transmission. At the national level, adopting the revised definition would provide a more accurate surveillance indicator of delayed diagnosis, but also require stronger routine capture and linkage of recent HIV acquisition markers and careful interpretation of time trends where definitions change. Future research should evaluate standardised clinical definitions of recent HIV acquisition to guide local reporting, help improve comparability between countries and assess implementation of the revised definition in routine national surveillance.

## Data Availability

Aggregated epidemiological HIV surveillance data are published by each country. Since the work was carried out using surveillance data information without individual patient consent as part of the legal requirement for public health surveillance and monitoring, the authors cannot make the underlying dataset publicly available for ethical and legal reasons. The code used for this study, which may be used to replicate the analysis, is available from: https://github.com/pkirwan/hivld-pilot-europe.

## Note

Part of this work has been presented at the EACS conference in October 2025 in Paris, France.

## Supplementary Data

934000 Supplement
