# Supplementary material for: Multi-country study on the impact of a revised consensus definition of late HIV diagnosis, Europe, 2022 to 2023
Source: Euro Surveill. 2026 Aug 13;31(32):2500921. doi: 10.2807/1560-7917.ES.2026.31.32.2500921 (PMC13476726; doi:10.2807/1560-7917.ES.2026.31.32.2500921)

Supplementary material for: Impact of a revised consensus definition of late HIV diagnosis in Europe: A multi-country study

This supplementary material is hosted by *Eurosurveillance* as supporting information alongside the article *Impact of a revised consensus definition of late HIV diagnosis in Europe: A multi-country study*, on behalf of the authors, who remain responsible for the accuracy and appropriateness of the content. The same standards for ethics, copyright, attributions and permissions as for the article apply. Supplements are not edited by *Eurosurveillance* and the journal is not responsible for the maintenance of any links or email addresses provided therein.

Supplementary Table S1: Specification for Multiple Imputation using Chained Equations, performed separately for each country.

**Variable name**	**Variable levels**	**Imputation method**
Age group*	15-24, 25-34, 35-44, 45-54, 55+	Ordered logistic regression
Gender	Male, Female	Logistic regression
Route of HIV transmission*	gbMSM, Non-gbMSM	Logistic regression
Region of origin	Reporting country, Sub-Saharan Africa, Latin America, Eastern Europe, Other	Multinomial logistic regression
Late diagnosis	Late/Not late	Logistic regression
Reclassification	Yes/No	Logistic regression

*Two or more variable levels were grouped to improve convergence: 55+ includes 55-64 and 65+; Non-gbMSM includes Heterosexual sex, PWID, and Other.

Supplementary Table S2: Number of new HIV diagnoses and availability of CD4 cell count information and recent acquisition evidence (any non-missing response) by country, year of HIV diagnosis, age group, gender, and route of HIV transmission. Among all nine study countries, 2022-23, n=15,924 new diagnosis records.

	**New HIV diagnoses**	**CD4 cell count within 90 days (%)**	**Laboratory evidence (%)**	**Last negative test evidence (%)**	**Clinical evidence (%)**	**At least one marker available (%)**
**Overall**	*15,924*	*64.2*	*32.3*	*7.8*	*39.7*	*62.8*
**Country**						
Belgium	*1,246*	*68.9*	*95.1*	*12.1*	*19.6*	*95.4*
Czechia	*502*	*83.7*	*100.0*	*7.8*	*3.4*	*100.0*
Denmark	*223*	*95.5*	*0.0*	*8.5*	*41.3*	*47.5*
Germany	*5,339*	*29.7*	*41.8*	*7.0*	*14.0*	*50.2*
Greece	*1,189*	*47.3*	*0.0*	*2.9*	*100.0*	*100.0*
Italy	*4,467*	*92.7*	*24.7*	*5.7*	*46.0*	*49.7*
Netherlands	*850*	*88.0*	*14.6*	*21.5*	*20.8*	*33.2*
Portugal	*1,784*	*77.2*	*0.0*	*7.9*	*95.0*	*95.3*

Sweden	*324*	*96.3*	*0.0*	*11.4*	*30.9*	*38.6*
**Year of HIV diagnosis**						
2022	*7,627*	*65.3*	*36.2*	*7.2*	*38.8*	*65.3*
2023	*8,215*	*63.8*	*29.0*	*8.2*	*40.7*	*60.8*
**Age group**						
15-24	*1,364*	*61.7*	*31.9*	*11.0*	*43.1*	*68.3*
25-34	*4,668*	*61.8*	*35.2*	*11.5*	*40.7*	*66.6*
35-44	*4,249*	*61.8*	*33.2*	*6.5*	*37.9*	*62.3*
45-54	*3,021*	*68.1*	*31.1*	*5.5*	*39.5*	*60.4*
55-64	*1,898*	*70.8*	*28.2*	*4.2*	*39.0*	*56.8*
65+	*681*	*68.4*	*23.5*	*4.0*	*41.4*	*56.7*
**Gender**						
Female	*3,772*	*61.5*	*33.9*	*2.9*	*36.5*	*62.7*
Male	*12,065*	*65.0*	*31.8*	*9.2*	*40.8*	*62.8*
Transgender	*67*	*80.6*	*46.3*	*29.9*	*22.4*	*68.7*
Unknown	*17*	*35.3*	*23.5*	*0.0*	*5.9*	*29.4*
**Route of HIV transmission**						
gbMSM	*6,397*	*70.3*	*33.7*	*14.7*	*45.7*	*67.9*
Heterosexual sex (Female)	*2,919*	*69.4*	*33.4*	*3.3*	*39.1*	*64.0*
Heterosexual sex (Male)	*2,564*	*82.1*	*27.6*	*3.2*	*44.0*	*61.0*
PWID	*771*	*52.1*	*31.1*	*5.1*	*41.1*	*65.6*
Other	*52*	*76.9*	*38.5*	*0.0*	*25.0*	*57.7*
Unknown	*3,189*	*35.2*	*32.1*	*2.3*	*24.7*	*52.0*
**Site of test**						
Community-based testing programme	*294*	*77.9*	*28.2*	*13.3*	*60.2*	*88.8*
Accident and emergency department	*285*	*90.2*	*0.4*	*7.7*	*96.8*	*97.9*
Infectious disease clinic	*269*	*92.6*	*30.5*	*11.2*	*57.6*	*87.7*
Primary health care	*775*	*79.4*	*12.5*	*10.2*	*62.6*	*72.1*
Sexual health or STI clinic	*449*	*78.2*	*25.2*	*28.5*	*42.8*	*69.7*
Other hospital setting	*660*	*87.9*	*19.2*	*4.2*	*43.8*	*61.2*
Other setting	*395*	*78.0*	*31.1*	*9.9*	*50.9*	*84.3*
Undetermined or unknown	*12,797*	*59.6*	*35.3*	*6.8*	*35.5*	*59.5*
**Region of origin**						
Same as country of report	*7,892*	*71.2*	*28.8*	*8.9*	*44.6*	*62.0*
Central Europe	*717*	*63.3*	*38.9*	*9.1*	*32.4*	*59.6*
East Europe	*1,184*	*56.9*	*43.1*	*4.4*	*21.7*	*60.0*
West Europe	*387*	*59.4*	*46.8*	*12.1*	*37.2*	*68.5*
North Africa and Middle East	*392*	*63.0*	*38.0*	*9.9*	*28.1*	*57.4*
Sub-Saharan Africa	*2,273*	*63.9*	*35.0*	*3.8*	*37.6*	*65.2*
Latin America and Caribbean	*1,445*	*67.5*	*27.0*	*11.6*	*46.5*	*66.1*
South and South East Asia	*445*	*65.8*	*29.0*	*6.7*	*28.3*	*50.1*
Abroad but sub-continent unknown	*117*	*62.4*	*28.2*	*12.0*	*35.0*	*54.7*
Unknown	*1,072*	*19.1*	*37.6*	*3.0*	*33.8*	*70.1*
**AIDS at diagnosis**						
Yes	*1,781*	*94.6*	*25.8*	*1.3*	*50.1*	*61.6*
No	*14,143*	*60.3*	*33.1*	*8.6*	*38.4*	*62.9*

Footnote: STI clinic: Sexually-transmitted infection clinic; gbMSM: gay, bisexual, and other men who have sex with men; PWID: People who inject drugs. “Other” routes of transmission include: mother-to-child, transfusion, nosocomial, and haemophiliac. Other hospital setting and other setting were reported by countries and may include: inpatient units, non-sexual health or STI outpatient clinics, home testing, and self-sampling. Having an unknown or undetermined exposure and/or site of test is usually due to data quality issues, rather than the information being truly unknown; national HIV surveillance systems may receive further information when individuals engage in care.

Supplementary Table S3: Hierarchy for different combinations of evidence, number of late diagnoses in each category (according to previous definition), and reclassification. Among all nine study countries, 2022-23, n=5,689 records with a CD4 count ≤350 or AIDS at diagnosis.

Value in hierarchy	Laboratory evidence	Last negative test evidence	Clinical evidence	Number of late diagnoses included	Recommendation to reclassify (Yes/No)
1	Yes	Yes	Yes	25	**Yes**
2	Yes	Yes	N/A	5	**Yes**
3	Yes	N/A	Yes	67	**Yes**
4	Yes	N/A	N/A	71	**Yes**
5	N/A	Yes	Yes	52	**Yes**
6	N/A	Yes	N/A	140	**Yes**
7	Not recent	Yes	Yes	11	**Yes**
8	Not recent	Yes	N/A	4	**Yes**
9	N/A	N/A	Yes	160	**Yes**
10	Not recent	N/A	Yes	27	**No***
11	N/A	N/A	N/A	4177	**No**
12	Not recent	N/A	N/A	950	**No**

Footnote: Yes: evidence suggests recent HIV acquisition, N/A: no evidence for recent HIV acquisition (N/A, missing, or not available), Not recent: avidity evidence indicates non-recent HIV acquisition. Nine study countries: Belgium, Czechia, Denmark, Germany, Greece, Italy, Netherlands, Portugal, and Sweden. *Indicates where the recommendation differs from the published definition.

Supplementary Table S4: Number of late diagnoses (according to previous definition) with evidence of recent acquisition, as reported by each country. Among all nine study countries, 2022-23, n=5,689 records with a CD4 count <350 or AIDS at diagnosis.

	Recent infection testing algorithm	p24 antigen	Positive PCR with negative/ indeterminate antibody test	Negative HIV test within 12 months	Primary HIV infection	Seroconversion illness
**Overall**	**78**	**60**	**74**	**237**	**35**	**307**
Belgium	0	24	24	28	22	56
Czechia	4	4	6	2	0	5
Denmark	0	0	0	7	6	0
Germany	29	0	0	36	0	109
Greece	0	0	0	7	0	6
Italy	45	5	10	72	0	64
Netherlands	0	27	34	35	0	47
Portugal	0	0	0	36	0	20
Sweden	0	0	0	14	7	0

Footnote: Totals exceed the number reclassified as diagnoses could be reported with multiple forms of evidence.

Supplementary Table S5: Proportion of values imputed for each variable in multiple imputation analysis, by reporting country. Among all nine study countries, 2022-23, and n=5,689 records with a CD4 count ≤350 or AIDS at diagnosis. Among all nine study countries, 2022-23, n=15,924 new diagnosis records.

	**Age group (%)**	**Gender (%)**	**Route of HIV transmission (%)**	**Country of origin (%)**	**Late diagnosis (%)**	**Reclassification markers (%)**
Belgium	0.2	2.5	25.3	20.1	31.1	4.6
Czechia	0.0	0.0	11.8	0.0	16.3	0.0
Denmark	0.0	0.0	10.3	2.7	4.5	52.5
Germany	0.7	0.1	32.3	8.6	70.3	49.8
Greece	0.0	0.3	27.8	22.3	52.7	0.0
Italy	0.0	0.0	10.4	0.7	7.3	50.3
Netherlands	0.0	3.3	14.6	0.8	12.0	66.8
Portugal	0.0	0.2	5.3	2.7	22.8	4.7
Sweden	0.3	4.3	16.0	0.9	3.7	61.4

Supplementary Figure S1: Demographic and site of test variables by route of HIV transmission. Among all nine study countries, 2022-23, n=15,924 new diagnosis records.

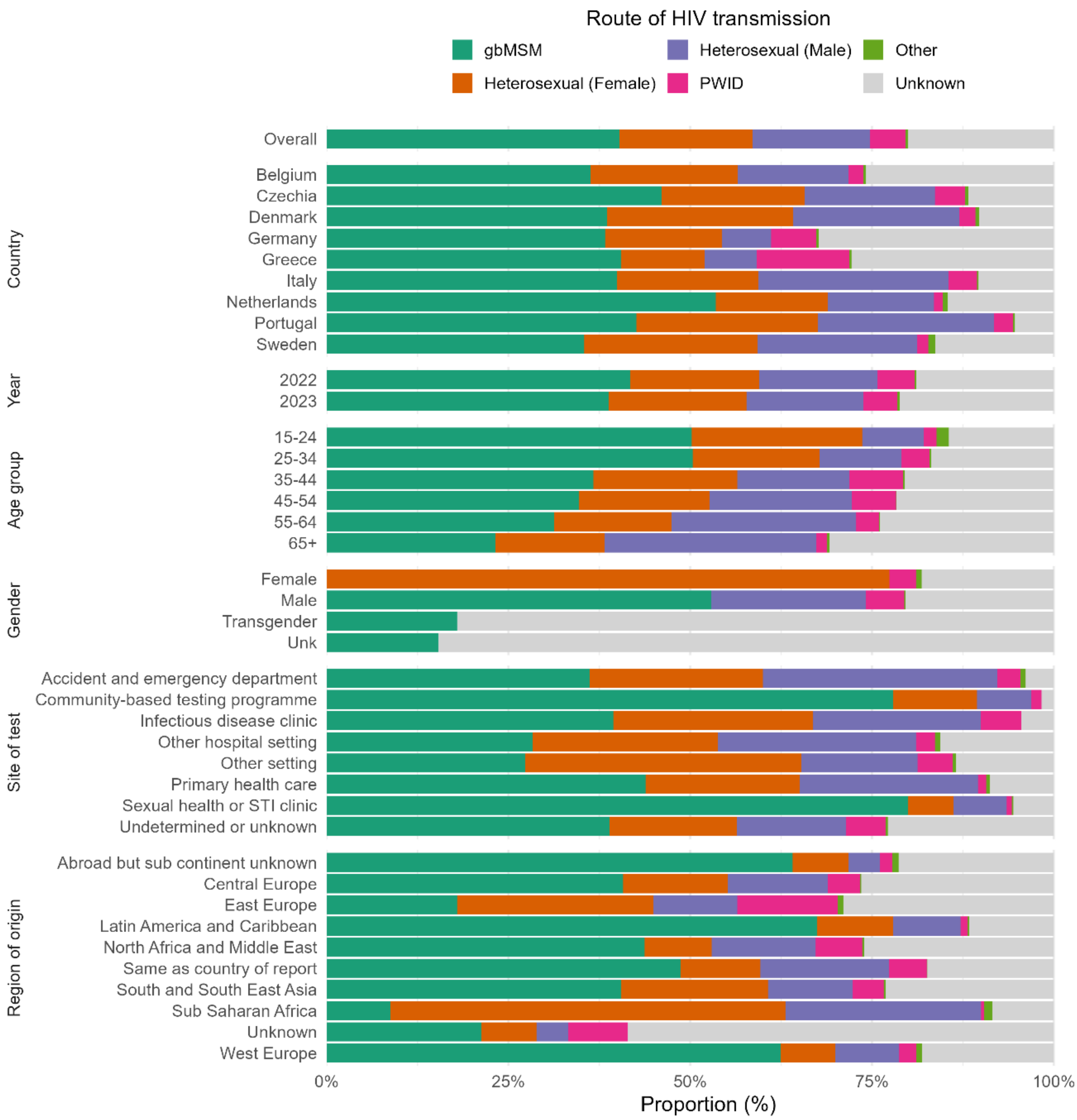

Supplementary Figure S2: Proportion diagnosed late before and after reclassification, by reporting country. Among all nine study countries, 2022-23, n=10,220 records with a CD4 count.

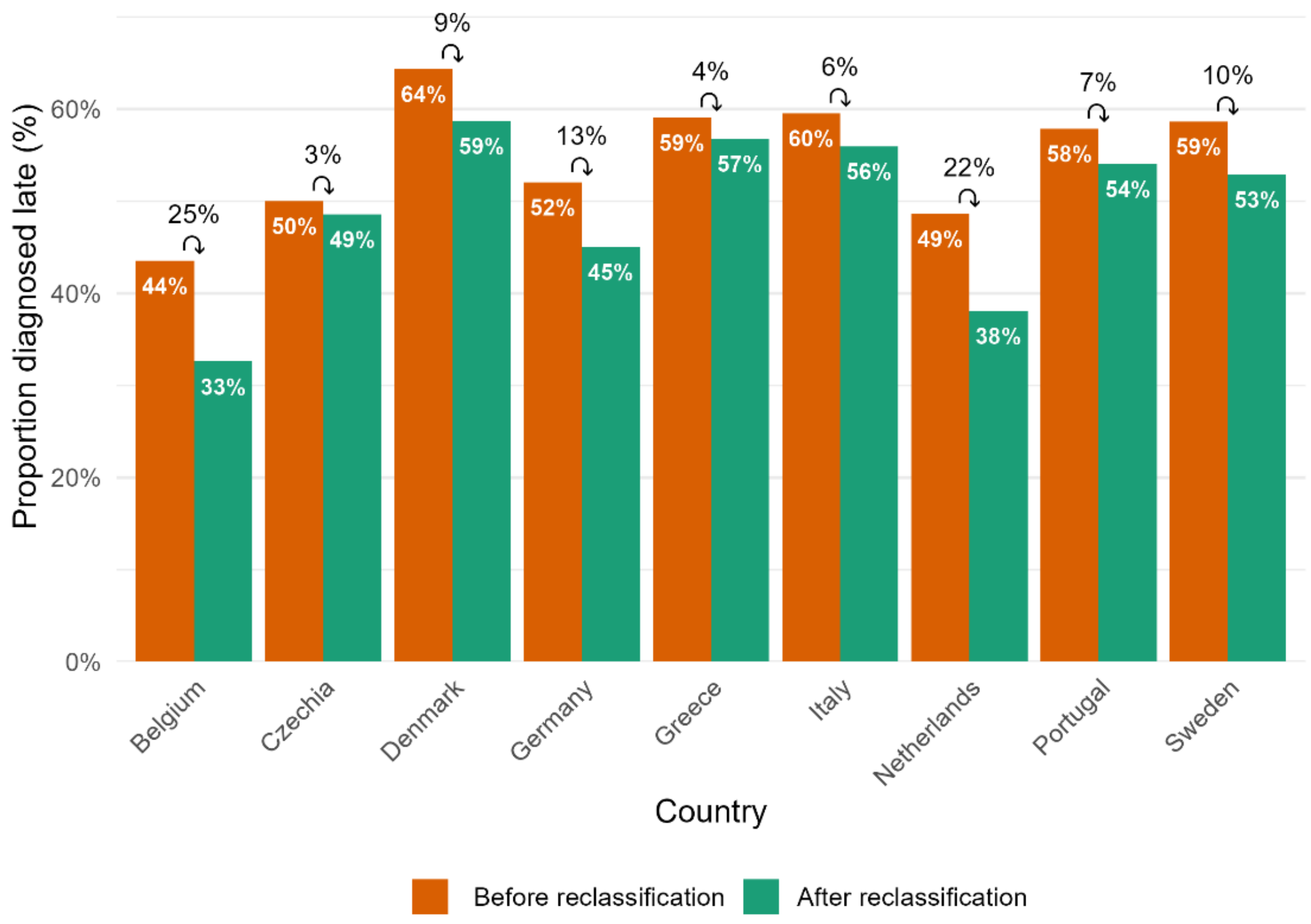

Footnote: Data labels are the proportion diagnosed late (within bars) and correction factor for each country (on top).

Supplementary Figure S3: Demographic and site of test variables by country of report. Among all nine study countries, 2022-23, n=15,924 new diagnosis records.

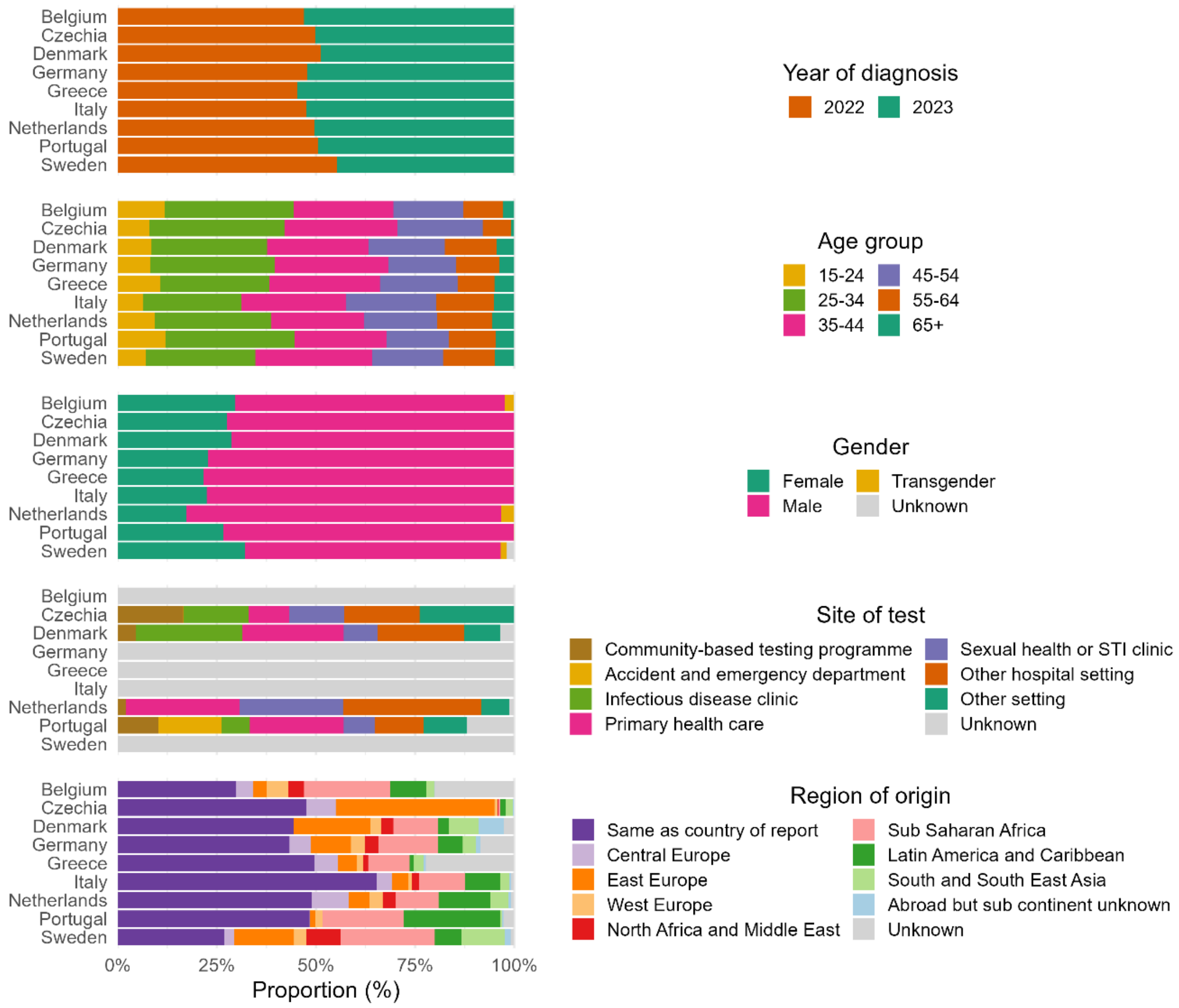

Study protocol

Piloting the revised HIV late diagnosis definition

A multi-country study coordinated under the EuroTEST Initiative

Study Coordinating Centre:
Secretariat of the EuroTEST Initiative
CHIP – Centre of Excellence for Health, Immunity and Infections
Rigshospitalet, University of Copenhagen, Capital Region of Denmark (RegionH)
Section 2100
Blegdamsvej 9
2100 Copenhagen Ø
Denmark
Phone: +45 35 45 57 57
Fax: +45 35 45 57 58
E-mail: chip@regionh.dk
Web: www.chip.dk

TABLE OF CONTENTS

1. BACKGROUND AND RATIONALE 3
1.1 Revised definition 3
2. STUDY AIM AND OBJECTIVES 3
3. STUDY PARTICIPANTS 4
3.1 Participating countries 4
3.2 Study subjects 4
4. METHODOLOGY 4
4.1 Study design 4
4.2 Datasets 4
4.3 Data collection and submission 4
5. STUDY PLAN 5
5.1 Statistical analysis 5
5.2 Limitations and validity considerations 6
5.3 Timeline 6
6. DATA HANDLING AND APPROVALS 6
6.1 Data confidentiality and handling 6
6.2 Regulatory approval 6
7. RISK AND ADVERSE EFFECTS 7
7.1 Risk and benefits 7
8. STUDY ADMINISTRATION 7
9. PUBLICATION OF STUDY RESULTS 7
10. LITERATURE 8
APPENDIX 1. LIST OF VARIABLES AND CODED VALUES 9
APPENDIX 2. LATE HIV DIAGNOSIS RECLASSIFICATION ALGORITHM 13

1. BACKGROUND AND RATIONALE

As testing for HIV has expanded and testing frequency increased across certain populations and regions in the European region, particularly among MSM and in relation to the roll out of pre-exposure prophylaxis (PrEP), some people may be diagnosed with HIV during the acute/seroconversion phase, when their CD4 count can be temporary low (known as the 'seroconversion effect').

In 2022, a EuroTEST working group revised the 2011 consensus definition of HIV late diagnosis [1] to enable better distinction between people diagnosed late from those who acquired HIV recently but were misclassified as late diagnoses due to a transient low CD4 cell count. The revised definition [2] includes markers of recent infection to reclassify those with a recently acquired HIV infection as not-late, with evidence of recent infection considered hierarchically.

This issue of overestimation has previously been raised by European research groups [3-5] and the updated definition is now routinely applied in several European countries [6-9].

1.1 Revised definition

Late HIV diagnosis is defined as a person first diagnosed with HIV with a CD4 count < 350 cells/µL or with an AIDS-defining event, regardless of the CD4 cell count. People with evidence of recent infection (i.e. being diagnosed during seroconversion) should be reclassified as 'not late'. Evidence of recent infection should be considered hierarchically; the individual must have: (i) laboratory evidence of recent infection [recent infection testing algorithm (RITA), p24 antigen]; (ii) a last negative HIV test within 12 months of HIV diagnosis; or (iii) clinical evidence of acute infection (e.g., seroconversion illness). People with evidence of having been previously diagnosed, either abroad or elsewhere, should be excluded from the calculation of the proportion of the population diagnosed late.

2. STUDY AIM AND OBJECTIVES

The aim of this multi-country pilot implementation study is to apply the revised definition of HIV late diagnosis to national HIV surveillance data [2]. This will involve collecting individual-level data on people newly diagnosed with HIV, and reclassifying people with a low CD4 cell count (<350 cells/ml blood) at diagnosis and evidence of recent infection to 'not late'. Country-specific 'recent infection correction factors' will be estimated, which will be applied to HIV surveillance data to adjust for the anticipated current overestimation of the proportion of people diagnosed late and provide updated estimates of late HIV diagnosis.

The pilot also seeks to generate experience from applying the definition in countries with different data availabilities for each of the required markers of recent infection: laboratory evidence of recent infection; last negative HIV tests within 12 months of HIV diagnosis; or clinical evidence of acute infection.

Further, ECDC will use the outcomes of the pilot to seek a consensus approach among the EU/EEA countries to modify the existing acute infection variable in TESSy and create a unified approach for the collection and analysis of HIV late diagnosis data.

The specific study objectives are to:

a) generate country specific estimates of the recent infection correction factors ('seroconversion effect') and apply these to existing surveillance data;
b) produce updated estimates of late HIV diagnosis in the participating countries;

c) propose analysis approaches and validity considerations for countries with differing availability of data on the various markers of recent infection; and
d) publish the results of the study and revised country-specific estimates in a peer-reviewed journal.

3. STUDY PARTICIPANTS

3.1 Participating countries

In 2022, as part of the process of updating the 2010 consensus definition of late HIV diagnosis, the EuroTEST working group conducted a survey among the 53 countries of the WHO European Region on the availability of data to monitor late HIV diagnosis and adjust for recent infection HIV diagnosis. Countries that met the following two criteria were initially invited to participate: a) expressed interested in participating by responding 'yes' to a survey question on interest in attempting to quantify the 'seroconversion effect' and corresponding correction factor for people diagnosed late in collaboration with EuroTEST; and b) had data available at national level for at least one of the three markers of recent infection.

Additional countries that are actively using (reporting on) and have data available for the variable 'AcuteInfection' in The European Surveillance System (TESSy) – the data collection tool used for the European level HIV surveillance conducted jointly by the European Centre for Disease Prevention and Control (ECDC) and the World Health Organization Regional Office for Europe (WHO/Europe) – were also invited to participate if more than 30 cases of acute infection were reported in the most recent reporting year (2022).

The following countries will participate in the study: Albania, Belgium, Denmark, Germany, Greece, Italy, Netherlands, Slovakia, Sweden. Participation by Czechia, Portugal and Switzerland is yet to be confirmed.

3.2 Study subjects

The study will be based on existing national surveillance registries of people newly diagnosed with HIV (case records from national surveillance). No new data collection or enrolment of individual study participants will take place for this study. No personal data that can identify the patient will be included.

4. METHODOLOGY

4.1 Study design

This is a retrospective analytical study based on existing national surveillance data with no independent participant enrolment.

4.2 Datasets

People aged 15 years or older (≥15 years) newly diagnosed with HIV in 2022 and 2023 in the participating countries will be included. People with evidence of having been previously diagnosed abroad ('previous positives') should be excluded. Case records will include information about age at HIV diagnosis (exact age or age category), gender, country of birth/region of origin and mode of HIV transmission (broad categories) but no personal identifiers will be collected, ensuring that the data are anonymous.

4.3 Data collection and submission

National datasets will be extracted from existing national surveillance registries by the country study coordinator on behalf of the participating country and submitted to the EuroTEST study coordinating centre

via a secure FTP server hosted at CHIP in Denmark. A pre-defined Excel reporting template with clear variable definitions will be made available to support dataset preparations.

The reporting template (Appendix 1) will include the following variables:

- Age at time of HIV diagnosis (exact age or age category), gender, mode of HIV transmission, date of HIV diagnosis (month/year if exact date is not available), date of AIDS diagnosis, date of death, first CD4 cell count, date of first CD4 cell count, country of birth/region of origin, place (setting) of first positive HIV test, laboratory evidence of recent infection, evidence of recent infection based on a negative HIV test within 12 months of HIV diagnosis and clinical evidence of acute infection (Appendix 1).

The required data fields and variable definitions will be aligned with the existing European level surveillance reporting format to reduce workload as much as possible.

The data transfer will take place via a SFTP (Secure File Transfer Protocol) server. SFTP is a file transfer protocol that delivers secure communications by encrypting both the transfer and the data itself. It ensures secure transfer of personal data and is approved for transferring patient sensitive data. Separate instructions for how to access and use the SFTP solution (available at https://sftp.regionh.dk) will be provided via email to each country study coordinator.

5. STUDY PLAN

5.1 Statistical analysis

The statistical analyses of the individual country datasets will be led by an experienced EuroTEST consultant in dialogue with the participating countries. The planned analyses will include:

1. descriptive statistics on the mode of HIV transmission and demographic characteristics of individuals diagnosed late in each country before correction;
2. calculation of the ‘recent infection correction factors’ according to mode of HIV acquisition and demographic characteristic; and
3. re-estimation of the late HIV diagnosis rate.

Since evidence of recent infection will be considered hierarchically, if multiple markers are available for a given individual, then the analysis will first use any laboratory evidence of recent infection, followed by the date of a last negative test, followed by any clinical evidence of acute infection. If no evidence of recent infection is available, then the individual will remain classified as a late diagnosis (see flowchart in Appendix 2).

The primary analysis will be to calculate country-specific estimates of the `recent infection correction factors’ (‘seroconversion effect’), resulting in revised estimates of the proportion of people diagnosed late with HIV in the participating countries. The recent infection correction factor is calculated as: the number of people diagnosed with a CD4 count < 350 cells/µL or with an AIDS-defining event reclassified as ‘not late’, divided by the total number of people diagnosed with a CD4 count < 350 cells/µL or with an AIDS-defining event.

The analysis will consider the number of people initially categorised as diagnosed late, alongside evidence of recent infection. The ‘recent infection correction factors’ will be assessed according to:

- Age, gender, and mode of HIV acquisition.
- Site of test and country/region of origin.

5.2 Limitations and validity considerations

The analysis approach may be limited by validity considerations for countries with differing availability of data on the various markers of recent infection and associated demographic information. For example:

- Missingness: if markers of recent infection are only available for a subset of cases in the national dataset, then additional assumptions or modelling may be required to impute missing information. A suggested completeness cut-off for valid imputation is 75%.
- National coverage: if data are only available from some regions of a country, then additional assumptions about representativeness of these data will be required, for discussion with each country.
- Availability of demographic information: sufficient completeness of demographic information will be needed to calculate the 'recent infection correction factors' for each sub-group.
- Number of diagnoses: where sub-groups are small, grouping of HIV transmission modes and/or age groups may be required to obtain meaningful estimates.

5.3 Timeline

- Summer 2024: Countries seek local ethics and/or legal (privacy) approvals
- Summer 2024: Data collation preparations (using provided reporting template)
- 31 August 2024: Data submissions to EuroTEST*
- Fall 2024: Data analysis and development of revised country level estimates
- Winter 2024-2025: Manuscript development

* Individual extensions into September may apply and will be agreed individually with each country upon request.

6. DATA HANDLING AND APPROVALS

6.1 Data confidentiality and handling

All data submitted to the study coordinating centre hosted at CHIP/Capital Region (RegionH) of Denmark are stored and protected in accordance with current regulatory laws and registered in Privacy according to article 30 in GDPR, with the local no: p-2024-16884. CHIP/Capital Region of Denmark (RegionH) strictly adheres to the General Data protection Regulation (GDPR) in Europe.

Case records collected in this study will include information about gender, age and mode of HIV acquisition but no other personal information. Each study subject ('case record') will have a simple identifier (a 'record ID) attached to it, constructed by each country for the purpose of participating in this study. If deemed useful and feasible by the country, this identifier could be identical to the record ID used for European level surveillance reporting. The record ID will be anonymous: no unique person identifiers nor any existing national de-coding lists will be shared with the study coordinating centre.

No individual level data will be included in any publication about this study.

6.2 Regulatory approval

It is the responsibility of each participating country to ensure that all necessary documents and approvals are obtained in accordance with national requirements and regulations. Sections from this protocol can be used if considered helpful by the given country.

7. RISK AND ADVERSE EFFECTS

7.1 Risk and benefits

Inclusion in the study does not include any risk for the participants. Study subjects are anonymous, and the study is purely analytical and based on existing data sources.

There are no direct benefits for participants. However, the study seeks to improve surveillance data on people diagnosed late with HIV by facilitating implementation of the revised definition of late diagnosis in the European countries and standardising European level reporting and analysis practices; thereby ensuring that the best possible public health information is made available to guide HIV prevention and testing programmes.

8. STUDY ADMINISTRATION

The study is coordinated under the EuroTEST Initiative whose secretariat is hosted at CHIP – an independent research institution at Rigshospitalet, Capital Region (RegionH) of Denmark, Copenhagen, Denmark.

The study is a continuation of work initiated in 2022 by a EuroTEST working group on HIV late diagnosis, resulting in publication of the revised consensus definition. The working group consists of independent experts from clinical, public health and community-based organisations across Europe [2].

The EuroTEST Initiative has received funding from ViiV Healthcare, Gilead Sciences and MSD-Merck. The funders have no input into the study design, analysis, decision to publish or content of the manuscript. National surveillance data are made available in-kind by the participating countries.

9. PUBLICATION OF STUDY RESULTS

The findings from this study are intended to be published in the peer-reviewed literature and/or be presented at relevant European conferences. Publications will be developed in accordance with internationally recognized scientific and ethical standards concerning publications and authorships and in agreement with the participating countries. The submitted data will solely be used for purpose of this project and will not be shared onwards.

All publications and presentations will be listed on the EuroTEST and CHIP webpages (www.eurotest.org and www.chip.dk).

10. LITERATURE

1. Antinori A, Coenen T, Costagiola D, Dedes N, Ellefson M, Gatell J, Girardi E, Johnson M, Kirk O, Lundgren J, Mocroft A, D'Arminio Monforte A, Phillips A, Raben D, Rockstroh JK, Sabin C, Sönnerborg A, De Wolf F; European Late Presenter Consensus Working Group. Late presentation of HIV infection: a consensus definition. HIV Med. 2011 Jan;12(1):61-4. doi: 10.1111/j.1468-1293.2010.00857.x.

2. Croxford S, Stengaard AR, Brännström J, Combs L, Dedes N, Girardi E, Grabar S, Kirk O, Kuchukhidze G, Lazarus JV, Noori T, Pharris A, Raben D, Rockstroh JK, Simões D, Sullivan AK, Van Beckhoven D, Delpech VC; EuroTEST HIV Late Diagnosis Definition Working Group. Late diagnosis of HIV: An updated consensus definition. HIV Med. 2022 Dec;23(11):1202-1208. doi: 10.1111/hiv.13425. Epub 2022 Nov 8.

3. Sasse A, Florence E, Pharris A, De Wit S, Lacor P, Van Beckhoven D, et al. Late presentation to HIV testing is overestimated when based on the consensus definition. HIV Med. 2016;17(3):231-4.

4. Brännström J, Svedhem Johansson V, Marrone G, Wendahl S, Yilmaz A, Blaxhult A, Sönnerborg A. Deficiencies in the health care system contribute to a high rate of late HIV diagnosis in Sweden. HIV Med. 2016;17(6):425-35. Epub 2015 Nov 11.

5. Tran TC, Cazein F, Le Strat Y, Barin F, Pillonel J, Lot F. Découvertes de séropositivité VIH chez les seniors en France, 2008-2016. Bull Epidémiol Hebd. 2018;(40-41):792-8. Available from: http://invs.santepubliquefrance.fr/beh/2018/40-41/2018_40-41_1.html

6. Statens Serum Institut. Hiv 2022 [internet]. [Cited 30 May 2024]. Available from: https://www.ssi.dk/sygdomme-beredskab-og-forskning/sygdomsovervaagning/h/hiv-2022

7. L'Istituto Superiore di Sanità. Aggiornamento Delle Nuove Diagnosi di Infezione da HIV E dei casi di AIDS in Italia al 31 Dicembre 2021. Rome: Istituto Superiore di Sanità; 2022.

8. Kirwan PD, Croxford S, Aghaizu A, Murphy G, Tosswill J, Brown AE, Delpech VC. Re-assessing the late HIV diagnosis surveillance definition in the era of increased and frequent testing. HIV Med [Internet]. 2022 Dec;23(11):1127–42. Available from: https://onlinelibrary.wiley.com/doi/10.1111/hiv.13394

9. Martin V, Okumu-Camerra K, Bera S, Shah A, Mackay N, Chau C, Ratna N, Aghaizu A, Humphreys C, Brown AE, and contributors. HIV Action Plan monitoring and evaluation framework 2023 report [Internet]. UK Health Security Agency, London; 2023 Dec [cited 2023 Dec 10]. Available from: https://www.gov.uk/government/publications/hiv-monitoring-and-evaluation-framework/hiv-action-plan-monitoring-and-evaluation-framework-2023-report

APPENDIX 1. LIST OF VARIABLES AND CODED VALUES

Variable	Full name	Description	Type	Coded value list
RecordId	RecordId	Unique identifier for each record within and across the national surveillance system – MS selected and generated	TEXT	
DateOfDiagnosis	Date of Diagnosis	The date of first HIV diagnosis; laboratory diagnosis. The exact date is preferred and should be provided if available; incomplete dates (e.g. quarter, month, year) are allowed if exact date is not available.	DATE	
DateOfAIDSDiagnosis	Date of AIDS diagnosis	Date of AIDS diagnosis. The exact date is preferred and should be provided if available; incomplete dates (e.g. week, quarter, month, year) are allowed if exact date is not available.	DATE	
DateOfDeath	Date of death	Date of death due to any cause. The exact date is preferred to obtain more accurate information and to allow better comparison and grouping. Incomplete dates (quarter, month, year) are permissible. All cases that are still alive or where the outcome (i.e., whether the case is alive or dead) is unknown are to be coded as 'NA'.	DATE	
FirstCD4Count	CD4 cell count at time of diagnosis	The variable specifies the CD4 cells count at the time of HIV diagnosis. Enter the numeric value of the CD4 (0-6000) or unknown (UNK).	NUM	
FirstCD4Date	Date of first CD4 cell count at time of diagnosis	The exact date is preferred and should be provided if available; incomplete dates (e.g. week, quarter, month, year) are allowed if exact date is not available. If FirstCD4Count is not available, enter NA for date.	DATE	

Age	Age	Exact age at diagnosis of HIV. Age as a crude number is preferred. If not available, age can be calculated by the MS from the date of birth and date of diagnosis.	NUM	
AgeCategory	Age	Age category at diagnosis of HIV. Alternative to reporting the exact age at diagnosis.	CV	AgeCategory:<br>1 = 15-19 years<br>2 = 20-24 years<br>3 = 25-29 years<br>4 = 30-34 years<br>5 = 35-39 years<br>6 = 40-44 years<br>7 = 45-49 years<br>8 = 50-54 years<br>9 = 55-59 years<br>10 = 60-64 years<br>11 = 65+ years
Gender	Gender	Gender of the reported case.	CV	Gender:<br>F = Female<br>M = Male<br>O = Other<br>T = Transgender<br>UNK = Unknown (default value)
Transmission	Describes the most probable route of Transmission	Most probable route of HIV transmission classified as: sex between men; heterosexual contact; ever injected drugs; other (mother-to-child transmission, transfusion recipient, nosocomial, haemophiliac). Nosocomial infection includes HIV acquired in health care settings. Cases of occupational exposure should be classified as exposure unknown or undetermined. Cases which are not fully documented should be coded as unknown.	CV	Transmission:<br>MSM = MSM/homo or bisexual male<br>HETERO = heterosexual contact<br>IDU = ever injected drugs<br>Other = MTCT (mother-to-child-transmission) OR NOSO (nosocomial) OR TRANSFU (transfusion recipient) OR HAEMO (haemophiliac patient).<br>UNK = Unknown or undetermined

SiteOfTest	Site of test	Describes the site of the first HIV reactive test for persons newly diagnosed with HIV, including screening tests prior to confirmatory testing performed outside of healthcare settings.	CV	SiteOfTest:<br>ABROAD = Tested abroad prior to arrival to reporting countryANS = Antenatal screeningBLOOD = Blood donation screeningCBT = Community-based testing programmeEMERG = Accident and emergency departmentINFDIS = Infectious disease clinic,OTHER = Other settingOTHHOSP = Other hospital settingPHARM = PharmacyPHC = Primary health carePRIS = Prison or remand servicesPWID = Harm reduction site/drug servicesSELFSAMP = Self-samplingSELFTEST = Self-testingSEXHEAL = Sexual health or STI clinicUNK = Undetermined or unknown
CountryOfBirth	Country of birth of patient	Defines the country of birth at country level, the ISO list of countries is provided. CountryofBirth is the preferred variable. If Unknown code as UNK.	CV	Country_Incl_HistCountries<br><br>(see the coded values list)
RegionOfOrigin	Region of origin of patient	Region from which the case originates. If the case is from the reporting country, it should be coded as REPCOUNTRY. CountryOfBirth is the prefered variable. If this is not available, then submit RegionOfOrigin. If both are submitted, CountryOfBirth should match RegionOfOrigin.	CV	RegionOfOrigin:<br>ABROAD = Abroad but sub-continent unknown<br>AUSTNZ = Australia and New Zealand<br>CAR = Caribbean<br>CENTEUR = Central Europe<br>EASTASIAPAC = East Asia and Pacific<br>EASTEUR = East Europe<br>EUROPE = If a case can not be reported in West, central or Eastern Europe, he/she should be reported in Europe (sub-continent unknown).<br>LATAM = Latin America<br>NORTHAFRMIDEAST = North Africa and Middle East<br>NORTHAM = North America<br>REPCOUNTRY = Same as country of report<br>SOUTHASIA = South and South East Asia<br>SUBAFR = Sub Sahara Africa

				Unk = Unknown<br>WESTEUR = West Europe
RecentInfectionLab	Evidence of recent infection based on laboratory evidence of recent infection [recent infection testing algorithm (RITA), or p24 antigen, or a positive PCR with a negative/ indeterminate antibody test]	An infection can be considered to be recent if a person has evidence from a p24 antigen assay, or a recency testing algorithm, or a positive PCR with a negative/indeterminate antibody test. This is a repeatable field and up to 3 options can be entered.	CV	RecentInfectionLab:<br>EV24ANT = Evidence from p24 antigen<br>RECTEST = Evidence from recency testing (RITA)<br>PCRAB = Evidence from a positive PCR with a negative/ indeterminate antibody test<br>NA = Not a recent infection based on laboratory evidence<br>UNK = Unknown
RecentInfectionNegTest	Evidence of recent infection based on last negative HIV test within 12 months of HIV diagnosis	An infection can be considered to be recent if a person has a negative HIV test within 12 months of diagnosis.	CV	RecentInfectionNegTest:<br>NEGTEST = Last negative test within 12 months of HIV diagnosis<br>NA = Not a recent infection based on negative test evidence<br>UNK = Unknown
RecentInfectionClinical	Evidence of recent infection based on clinical evidence of acute infection (e.g. seroconversion illness and/or evidence of primary HIV infection)	An infection can be considered to be recent if a person presents with seroconversion illness (based on clinical judgement of symptoms and medical history) and/or evidence of primary HIV infection (PHI) (high-risk exposure within previous 6 weeks, and a) detectable virus in plasma (p24 Ag and/or HIV-RNA) and/or b) evolving anti-HIV antibody reactivity (negative or indeterminate to positive) – with or without clinical symptoms). It is preferred to report laboratory and clinical evidence separately (as the markers are considered hierarchically in the revised definition) but PHI can be reported by countries that use this definition nationally.	CV	RecentInfectionClinical:<br>SEROILL = Seroconversion illness<br>PHI = Primary HIV infection<br>NA = No a recent infection based on clinical evidence<br>UNK = Unknown

APPENDIX 2. LATE HIV DIAGNOSIS RECLASSIFICATION ALGORITHM

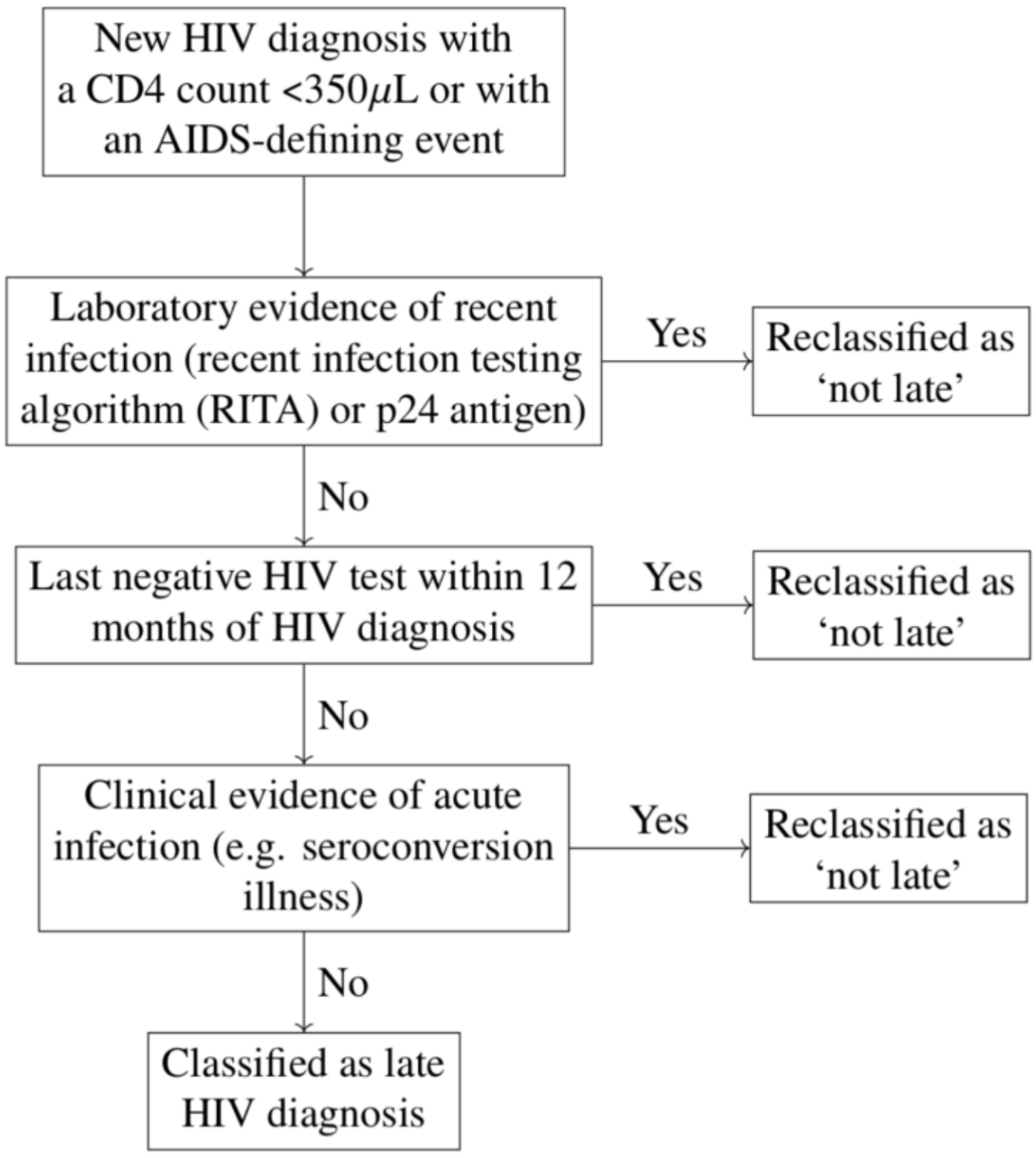

Supplement: Supplementary file 1 [file 25-00921_KIRWAN_Supplementl.pdf]
